# Retroperitoneal Solitary Fibrous Tumor: A “Patternless” Tumor

**DOI:** 10.1155/2017/4634235

**Published:** 2017-09-12

**Authors:** D. Myoteri, D. Dellaportas, C. Nastos, I. Gioti, G. Gkiokas, E. Carvounis, T. Theodosopoulos

**Affiliations:** ^1^Pathology Department, Aretaieion University Hospital, Medical School of Athens, Athens, Greece; ^2^2nd Department of Surgery, Aretaieion University Hospital, Medical School of Athens, Athens, Greece

## Abstract

**Introduction:**

Solitary fibrous tumor is a rare type of mesenchymal, spindle-cell tumor reported mostly in the pleura. Retroperitoneal occurrence is rare and histopathological diagnosis is challenging.

**Case Presentation:**

A 55-year-old woman with nonspecific abdominal pain was found to have a retroperitoneal/pelvic mass adjacent to the upper rectum. The patient underwent surgical resection in clear margins of this pelvic tumor, entering the total mesenteric excision surgical plane. Final histopathology revealed a solitary fibrous tumor and the case is presented herein.

**Discussion:**

Solitary fibrous tumor in the retroperitoneum is rarely found in the literature and to the best of our knowledge less than a hundred cases are described so far. Histopathological diagnosis is mostly based on a “patternless pattern” on microscopic examination, which is a storiform arrangement of spindle cells combined with a “hemangiopericytoma-like appearance” and increased vascularity of the lesion. Surgery is the mainstay of treatment and recurrence rates are generally low.

## 1. Introduction

Solitary fibrous tumor (SFT) is a rare type of mesenchymal, spindle-cell tumor and includes heterogenous variety of neoplasms, both benign and malignant. They are mostly reported arising in the pleura, and about 30% develop in extrapleural tissues, including the retroperitoneal space [1]. Surgical resection in clear margins is the mainstay of treatment, alike all retroperitoneal sarcomas. Histopathological diagnosis is very interesting and stepwise, based on exclusion criteria and on a characteristic “patternless pattern” of the spindle cells. A rare case of a retroperitoneal SFT is presented herein, along with the histopathological and oncological challenges of this seldom found tumor [2].

## 2. Case Presentation

**Figure 1:**
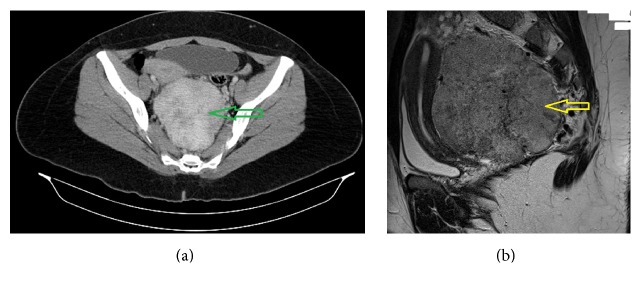
(a) Computed tomography (CT) of the abdomen. Green arrow showing the large pelvic mass. (b) Sagittal view-Magnetic Resonance Imaging (MRI). Yellow arrow showing the tumor.

A 55-year-old woman was investigated for nonspecific lower abdominal and back pain. The patient's past medical and surgical history was clear and physical examination as well as routine haematological and biochemical laboratory investigations were unremarkable. Computed tomography (CT) of the abdomen and pelvis revealed an approximately 10 × 10 cm tumor in the retroperitoneal space, immediately anterior to the aortic bifurcation, high in the pelvis, and posteriorly to the upper third of the rectum (Figure 1(a)). Magnetic resonance imaging (MRI) of the pelvis followed and confirmed the solid nature of this mass, showing that it was independent from the bowel/rectum, featuring a retroperitoneal sarcoma type of mass rather than a lymph nodal bloc (Figure 1(b)).

**Figure 2:**
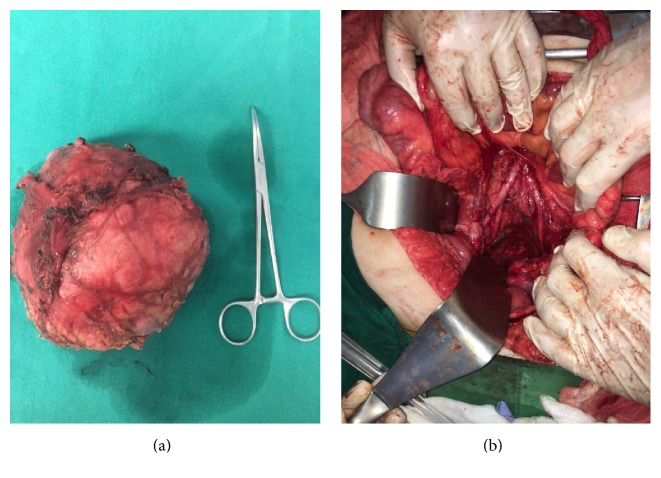
(a) Macroscopic view of the surgical specimen. (b) Intraoperative view of the pelvis, after tumor resection. Total mesenteric excision plane was entered.

After multidisciplinary team (MDT) meeting discussion, surgical exploration was decided and performed via midline laparotomy. Bilateral ureter guidewires were inserted intraoperatively, to facilitate identification of the ureters. The left colon was mobilized and the total mesorectal excision (TME) surgical plane was entered; the tumor was mobilized and excised in clear margins macroscopically without any intraoperative adverse events (Figures 2(a) and 2(b)). There was no close relation of the tumor to any adjacent anatomical structure, having its blood supply from small arterial branches originating from the common iliac arteries and the mesentery of the rectum. The mass was resected without compromising the integrity of the rectum or the sigmoid colon, excluding any relationship with the uterus as well. The patient had an uneventful postoperative course and was discharged on the 7th day postop.

**Figure 3:**
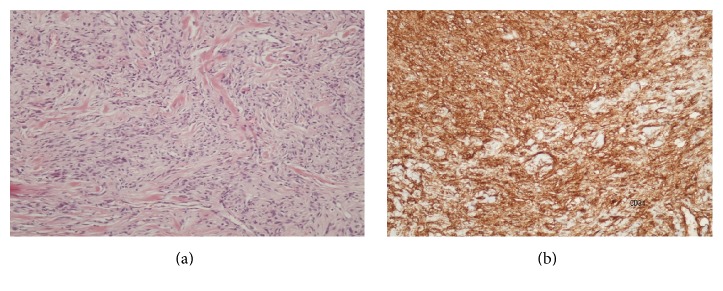
(a) Microscopic view (H-E ×100) of bland and uniform oval to spindle cells with minimal cytoplasm, small elongated nuclei, and indistinct nucleoli. (b) Immunohistochemical examination with positive staining for CD34.

Histopathological examination showed a neoplasm composed of bland and uniform oval to spindle cells with minimal cytoplasm, small elongated nuclei, and indistinct nucleoli (Figure 3(a)). The tumor exhibited an overall patternless architecture of hypo- and hypercellular areas separated by thick, hyalinized collagen with cracking artifact and staghorn vessels. The neoplasm had minimal pleomorphism, no atypia, and rare mitotic figures (<1 mitoses per 10 High Power Fields). Neither necrosis nor hemorrhagic alterations were observed. Immunohistochemical examination showed positive staining for Bcl-2, CD34 (Figure 3(b)), vimentin, and CD99 while desmin and S-100 were negative. The Ki-67 index was 7%, confirming the overall indolent nature of this tumor. Final diagnosis was retroperitoneal solitary fibrous tumor.

No adjuvant treatment was decided on the MDT and the patient remains asymptomatic and tumor free on follow-up visits one year later.

## 3. Discussion

SFTs are soft tissue spindle-cell neoplasms, first described by Klemperer and Rabin in 1931 [3]. World Health Organization (WHO) classifies SFT as intermediate fibroblastic or myofibroblastic tumors along with hemangiopericytomas, which means that SFTs are considered tumors that rarely if ever metastasize [4]. These neoplasms usually affect the pleura, while extrapleural sites are reported in about 30% of cases. The latter include the nasal cavity, salivary glands, orbit, upper respiratory tract, thyroid, peritoneum, genitourinary system, and retroperitoneum and pelvis [5].

SFT in the retroperitoneum, as in the case reported above, is rarely found in the literature and to the best of our knowledge less than 100 cases are described so far [6]. The main characteristic of these is the large size they can reach due to the lack of specific symptoms, leading to the need for major surgical resections of the primary tumor along with adjacent structures.

Histopathological diagnosis is challenging and mostly based on a “patternless pattern” on microscopic examination. This pattern is a storiform arrangement of spindle cells combined with a “hemangiopericytoma-like appearance” and increased vascularity of the lesion [7]. Differential diagnosis includes other spindle-cell neoplasms such as leiomyoma, inflammatory myofibroblastic tumor, angiomyolipoma, and gastrointestinal stromal tumor. Immunohistochemistry is very helpful, and SFTs are positive for Bcl-2, vimentin, and CD99, as well as CD34 [8]. Negative expression of S100, cytokeratin, EMA, SMA, CD117, CD31, and desmin is the norm and adds to the correct diagnosis. The combination of positive Bcl-2 and CD34 is guiding histopathologically towards the diagnosis of SFT, since 75% of extrapleural SFTs positively express these two markers [5].

SFTs are considered malignant when histopathological examination shows high cellularity, high mitotic activity (more than 4 mitoses per 10 HPF), pleomorphism, necrosis, and hemorrhagic changes [9].

Moreover, SFTs can cause paraneoplastic syndromes and mainly hypoglycemia, which is thought to arise due to the production of Insulin like Growth Factor-2 (IGF-2) from the tumor. Such a condition may be the presenting symptom for these cases [10]. When complete resection is feasible these syndromes subside. This was not the case for the patient presented above.

Tomographic imaging cannot differentiate retroperitoneal SFT from other solid retroperitoneal sarcomas [11, 12]; however it is invaluable to guide the surgical team to the right approach and strategy for radical excision in clear margins. As with most of the sarcoma-like tumors, surgery is the main and usually the only effective treatment of SFTs. In the whole, recurrence rates are low and positive resection margins seem to affect these rates [13], underlying the importance of a sound surgical excision.

Due to the rarity of SFTs, especially in the retroperitoneum, studies to define the best management approach are lacking and adjuvant treatment options are based on case reports and observational studies. Interestingly, antiangiogenic drugs, as bevacizumab, based on the high vascularity of the lesion are used initially and conventional chemotherapy to keep the disease stable is a strategy proposed in an important study on the matter, treating advanced disease [14]. Even benign cases are reported recurring locally or at distant sites, indicating unpredictable behavior of this rare neoplasm, with malignant transformation potential [15]. The latter is the basis of follow-up with tomographic imaging.

In conclusion, SFT in the retroperitoneum should be managed aggressively with primary surgery and has a good prognosis. Histopathological diagnosis is stepwise and immunohistochemistry can guide towards the right direction in equivocal cases. Uncertain clinical behavior and lack of management guidelines confuse clinicians and multidisciplinary team approach is of paramount importance.
